# Chronic Avulsion Lesion at the Brachialis Muscle Insertion

**DOI:** 10.5334/jbsr.2616

**Published:** 2021-11-19

**Authors:** Dima Al Jahed, Filip Vanhoenacker

**Affiliations:** 1 AZ Sint-Maarten, Mechelen Faculty of Medicine and Pharmacy, University of Brussels, Brussels, BE; 2 AZ Sint-Maarten and University (Hospital) Antwerp/Ghent, BE

**Keywords:** Humeral splints, brachialis muscle, magnetic resonance imaging, computed tomography

## Abstract

**Teaching Point:** Humeral splints or brachialis insertion avulsion syndrome is a distinct type of stress injury at the insertion of the brachialis muscle on the lateral surface of the humeral diaphysis.

## Case Presentation

A 26-year-old man presented with pain in the lateral side of the left upper arm. The patient was known with multiple sclerosis for which he has been doing weight training to preserve muscle strength.

**Figure 1:**
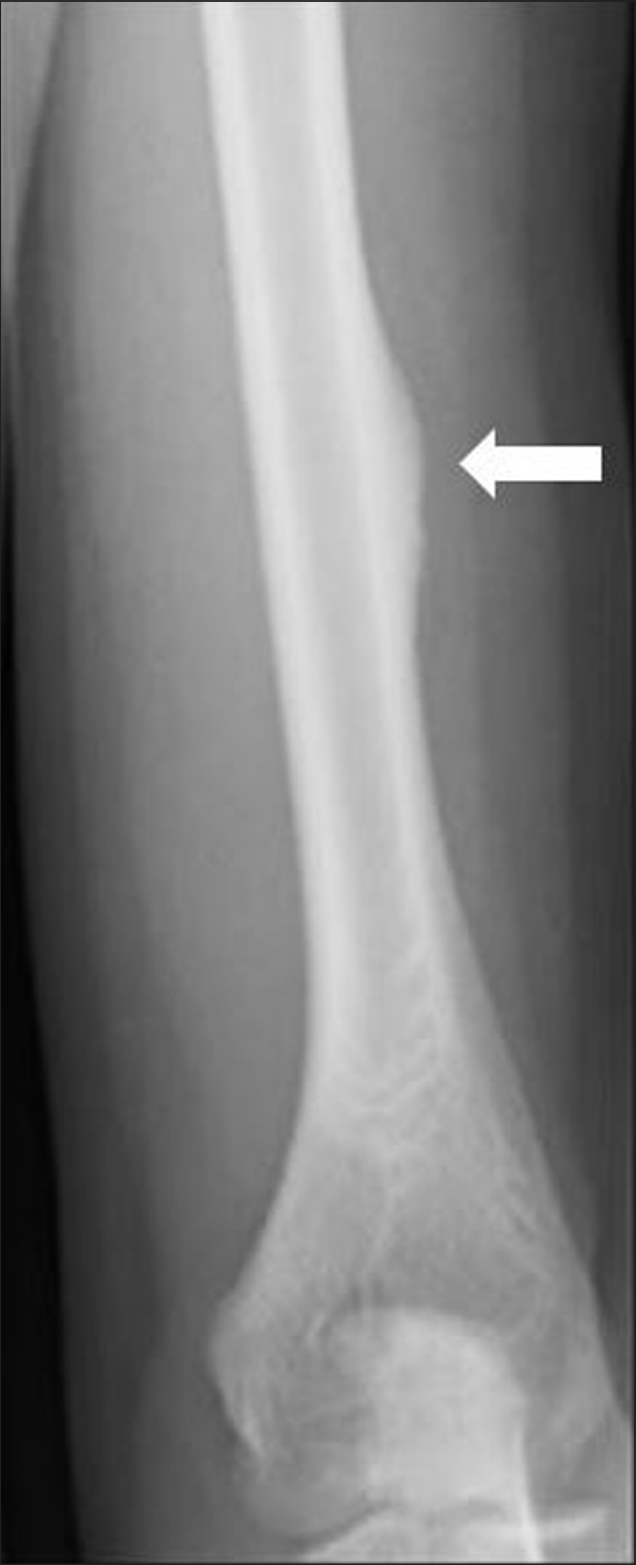


Plain radiograph of the left humerus showed cortical thickening along the lateral aspect of the distal third of the left humeral diaphysis (***Figure 1***, arrow).

**Figure 2:**
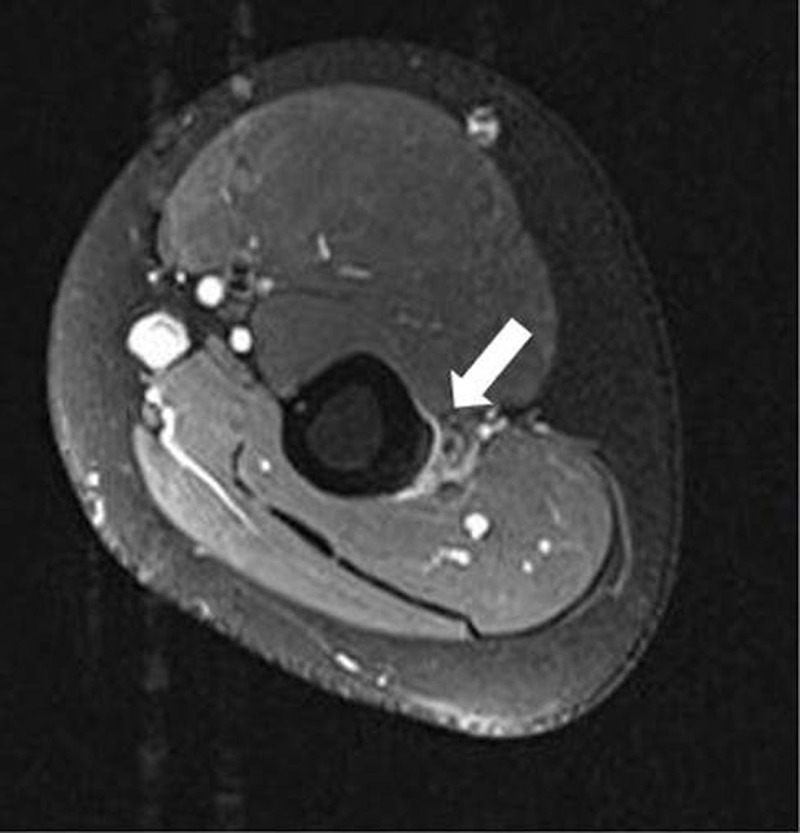


Magnetic resonance imaging (MRI) revealed a linear area of high signal intensity at the periosteal side of the humeral shaft as well as adjacent muscle oedema at the insertion of the brachialis muscle on Fat suppressed (FS) T2-weighted image (WI) (***Figure 2***, arrow).

**Figure 3:**
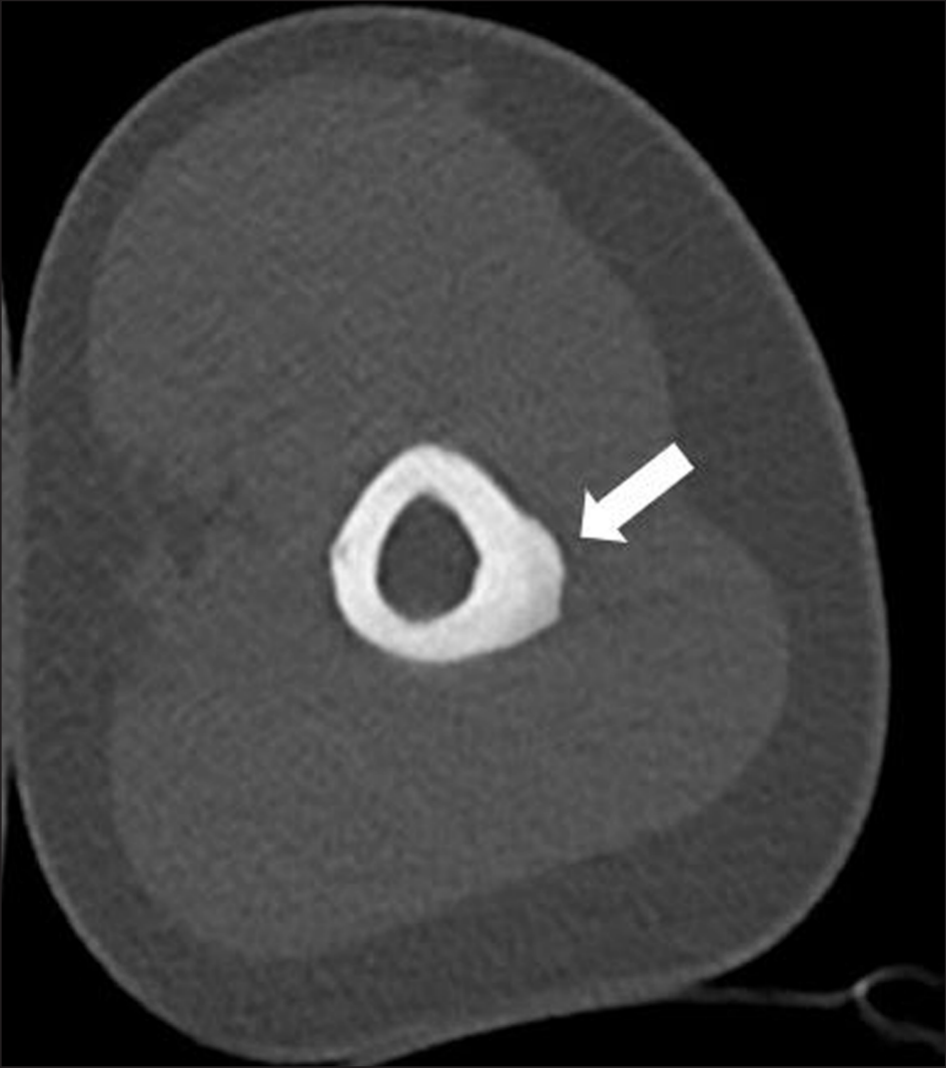


Subsequent computed tomography (CT) excluded an osteoid osteoma and confirmed focal cortical thickening with solid periosteal reaction (***Figure 3***, arrow).

Based on these imaging findings the diagnosis of humeral splints was made.

## Discussion

Humeral splints or chronic avulsion fracture of the brachialis muscle insertion on the humerus is a rare injury that usually relates to weightlifting sports. It is a lesser-known type of stress injury, similar to shin splints of the tibia or thigh splints at the femur.

The pathogenesis is still not yet fully elucidated. As it most commonly involves athletic adolescents, it is widely believed to be caused by repetitive chronic avulsive stress at tendinous insertion of the brachialis muscle, leading to increased tension and traction periostitis. In our case, the lesion is attributed to the intensive weight training. The resulting changes in the bone may range from an asymptomatic focus of accelerated bone remodelling to an overt stress fracture.

Conventional radiographs are often normal, in case of subtle lesions but cortical thickening containing hyperlucent areas with a periosteal reaction at the distal lateral humeral shaft may be seen [1].

MRI shows increased signal intensity on T2-WI along the periosteum of the lateral side at the humeral diaphysis with subtle edema in the brachialis muscle. There is absence of visible intracortical fracture lines nor cortical destruction or adjacent soft tissue mass [1].

Cross-sectional imaging is very useful to rule out osteomyelitis, eosinophilic granuloma, osteogenic sarcoma, or Ewing’s sarcoma. CT is useful to rule out osteoid osteoma.

The typical location of the lesion at the insertion of the brachialis muscle is the clue to the diagnosis. It should be differentiated from a pseudotumor deltoideus or chronic avulsion injury of the deltoid insertion which is located more proximally.

Correct diagnosis may avoid unnecessary biopsy. Usually, pain resolves after rest and conservative treatment [1].
